# The Role of Nurse Leaders in Maintaining Healthy Work Environments for Nurses in a Mental Health Hospital: A Qualitative Study

**DOI:** 10.1155/jonm/5598476

**Published:** 2025-09-12

**Authors:** Mohammed Almalki, Bayan Alilyyani, Fahad M. Althobaiti, Nawaf Alzahrani, Eyad Al-Talhi, Riyadh Alharthi, Wesam Alghamdi, Abdulrahman Al-Otaibi, Shadi Al-Shamrani

**Affiliations:** Department of Nursing Management and Education, College of Nursing, Taif University, Taif 2425, Saudi Arabia

**Keywords:** environment, hospital, interview, leader, mental, nurse, qualitative

## Abstract

**Background:** Dealing with the complex psychological aspects of patient care required a good leader to maintain healthy work environments. Even early in a nursing career, the call for leadership was an essential aspect to enhance the outcomes related to both nurses and patients, especially in the challenging field of mental health nursing. It is known that a supportive work environment is important for nurses working in mental healthcare settings.

**Purpose:** The purpose of this study was to explore the role of nurse leaders in maintaining healthy work environments for nurses working in a mental health hospital in Taif, Saudi Arabia.

**Methods:** This study used a descriptive-qualitative design. Data were collected from nurse leaders at the Mental Health Hospital in Taif City, Saudi Arabia. Nonprobability, purposive sampling was applied to collect data. Data were collected through semistructured interviews. NVivo was used as a software management system to analyze the data.

**Results:** Nine nurse leaders participated in this study. Thematic analysis was applied to determine the categories of narrative analysis. Based on the participants' interviews, the findings were categorized into two main themes and six subthemes which are Facilitators to Maintain Healthy Work Environments: *Ways to Maintain Healthy Work Environments, Building Teamwork, The Required Leadership Skills in Mental Health Hospitals, Entertainment;* and Barriers to Keep the Environments Healthy: *Mental Health Nurses' Traits, and Burnout*.

**Conclusion:** Nurse leaders have an important role in maintaining a healthy work environment for mental health nurses, decreasing stress and workload, and improving nurses' outcomes. Nurse leaders also have to pay attention to their staff's mental health if they are in leadership positions in mental healthcare hospitals.

## 1. Introduction

Nurses are one of the essential healthcare teams who are responsible for delivering high-quality treatment and guaranteeing favorable patient outcomes. The complex nature of mental health disorders presented unique challenges for nurses working in the specialized setting of mental health hospitals [1]. In order to preserve nurses' well-being and improve patient care, the demanding work environment and complex patient needs require strong leadership and supportive structures [2]. Mental health was viewed as a basic human need that negatively impacted every person's quality of life. In recent years, there has been a growing awareness of the critical role of nurse leaders in creating and maintaining a positive work environment for nurses in mental health hospitals [3]. According to Montano et al. [4], emotional, psychological, and social well-being are considered as different forms of mental health. Taking into account all aspects of mental health, maintaining it in a work environment was no longer viewed as merely an individual employee responsibility. Instead, leaders have the duty to maintain the mental health of their staff members [5].

There was a recent review conducted by Stuber et al. [6] to investigate the knowledge regarding nurse leaders' contributions to the development of a positive work environment for mental health staff. It was found that leaders have the ability to keep the work climate healthy for nurses and motivate them to provide the best quality of care for mental patients [6]. Effective leadership and efficient communication were found as key elements that made up a positive work environment for staff [7]. Mental health nurses who worked in supportive environments were less likely to experience work environment stressors [7]. Leaders' approaches, tactics, and leadership styles have an impact on staff retention rates, work satisfaction, and standard of care provided to mental patients [8]. Nurses who worked in units where nurse leaders are supportive showed higher levels of responsibility [9].

Mental health nurses expressed a high intention to leave their jobs, compared to general hospital nurses [10]. In addition, mental health nurses who are not able to attain a high degree of work engagement could have an impact on the quality of care provided to their patients [11]. Thus, it is essential to comprehend the viewpoints and experiences of nurse leaders in mental health hospitals in order to devise successful tactics that fostered a positive work environment by using qualitative study designs. Qualitative studies offered a thorough examination of the perceptions, practices, and experiences of nurse leaders. They also provided important insights into the difficulties they encountered and the methods they used to establish a supportive work environment for their nursing staff.

This study sought to explore the role of nurse leaders in preserving a healthy work environment for nurses in a mental health hospital. This study would offer important insights that could aid in the creation of practical tactics and interventions to support nursing staff and enhance patient care in the field of mental health nursing by looking at the experiences and methods of nurse leaders. This study would also add to the body of knowledge already in existence regarding the function of nurse leaders in preserving a positive work environment for nurses in mental health hospitals by combining the results of qualitative studies. It also provides insights for future research directions and practical implications for nurse leaders and policymakers seeking to improve the work environments in mental health settings.

### 1.1. Research Question

What are the roles of nurse leaders in maintaining a healthy work environment for nurses in a mental health hospital?

## 2. Materials and Methods

### 2.1. Design

A descriptive-qualitative design was used to explore the role of nurse leaders in maintaining healthy work environments for nurses working in a mental health hospital.

### 2.2. Study Settings

Data were collected from nurse leaders at the Mental Health Hospital in Taif City, Saudi Arabia. The hospital is public and operated by the Saudi Ministry of Health. The bed capacity of the hospital is around 700 beds. It provides services in inpatient and outpatient departments. Also, the hospital has an emergency department to provide services all the time.

### 2.3. Participants

In this study, nonprobability, purposive sampling was used. The criteria for participation in the study included being nurse leaders including head nurses, charge nurses, nurse supervisors, and nurse directors with experience in leading nurses in mental health settings for more than 2 years and having worked in an adult inpatient mental health hospital. However, registered nurses or nurse leaders with no experience were excluded. An estimated number of participants were from 8 to 10 nurse leaders. Data saturation was reached by participant number 9.

### 2.4. Data Collection

After obtaining the ethical approval, the hospital administrators facilitated the data collection for researchers. They provided the researchers with the number of nurse leaders in the hospital and contact information to reach out to them. Researchers then contacted the nurse leaders, who agreed to participate in the study. The interviews took place in the hospital where the nurse leaders were employed, and the information was transcribed after being captured on audio. Data were collected through semistructured interviews. Eight open-ended questions were used to explore the role of nurse leaders in maintaining a healthy work environment for staff nurses, the best ways to enhance the leadership skills of mental health nurse leaders within the work environment, the barriers they faced, and the impact of education and/or experience in enhancing their leadership skills. Interviews were conducted by the researchers and were audio recorded. Each participant interview lasted from 40 to 60 min.

### 2.5. Data Analysis

During the planning phase, thematic analysis was chosen [12]. For this purpose, the written files were read multiple times to identify the sentences and matching codes by the five researchers. After that, two other researchers reviewed the codes to minimize bias. If there is disagreement in the coding process, the research team meets to discuss and ensure the accuracy of the coding process. Throughout the arranging stage, categories were formed, and the retrieved codes from the previous stage were continuously compared. Once the categories were named, the idea map was chosen, and thematic analysis was applied. NVivo was used as a software management system to analyze the data.

### 2.6. Ethical Considerations

Ethical approval for the study was obtained from Health Affairs in Taif (IPR NO: 763, Date: 23/12/2022). No actions were taken related to the study before obtaining the approval. Agreeing to be interviewed was considered a voluntary agreement. A consent form was used before interviewing the participants. Personal information was collected only for the consent form, but it was not read by anyone other than the research team, and the information of any of the participants or any private details that were not related to the essence of the research were not written about. Additionally, the participant had the right not to participate or to withdraw from the study at any stage.

### 2.7. Rigour

According to accepted standards for qualitative research, trustworthiness to guarantee rigour in the findings was researchers' goal [13, 14]. The fact that nurse leaders from each department were chosen to participate in all interviews increased the rigour by having participants consider each interview and transcribe all the content. The head nurses' leadership experience and diverse work experience in mental care settings proved beneficial for the analytical process. Credibility, transferability, dependability, and confirmability were applied to ensure the rigor and trustworthiness in this study. To ensure credibility, researchers during the interviews focused on the main phenomenon and spent enough time with each participant. Also, they used semistructured interviews to collect data in depth. Transferability was ensured by using a clear method in terms of design, sample, data collection, and analysis. Dependability was certified by stability of this study's findings. The results of this study can benefit other researchers, who can use it as a reference in the future to conduct studies. Also, nurse leaders could apply the results of this study to enhance the mental health settings. Finally, researchers applied different mechanisms to ensure the confirmability of this study. Researchers discussed the disagreement, if any, to minimize the bias. In addition, two researchers reviewed the coding process. They also shared the findings with participants to ensure the accuracy of results.

## 3. Results

### 3.1. Demographics of Participants

The number of participants who met the inclusion criteria was nine. The details of the participants are illustrated in Table 1. Eight of nine were heads of departments, and six were Saudi. The mean age of the participants was 37 years old. More than half of the participants were male.

### 3.2. Narrative Analysis

Based on the participants' interviews, the findings were categorized into two main themes and six subthemes which are Facilitators to Maintain Healthy Work Environments: *Ways to Maintain Healthy Work Environments, Building Teamwork, The Required Leadership Skills in Mental Health Hospitals, Entertainment;* and Barriers to Keep the Environments Healthy: *Mental Health Nurses' Traits, and Burnout*.  Table 2 illustrates the findings' themes and subthemes.

### 3.3. Facilitators to Maintain Healthy Work Environments

#### 3.3.1. Ways to Maintain Healthy Work Environments

Seven out of nine nurse leaders reported that the environment in mental health hospitals is radically different from other healthcare settings, which makes mental health hospitals differ in their environments from others. In addition, the needs, requirements, and skills that a nurse should have in mental health hospitals are different from the requirements, skills, and needs that a nurse should have in any other hospitals. For example, one of the requirements of mental health hospitals is that they need more male nursing staff, while some other departments need qualified nurses who have taken specific courses or certificates. The hospital environment is considered psychologically tiring and mentally stressful for nurses because of the types of patients are dealt with directly. Also, nurses have to deal with the unprepared environment and must be aware and anticipate any movement or attack from patients.

The environment should be prepared for any situations in the department, and nurses should know how to care of their patients. One of the head nurses said that “*The counter must be located in the center and protected by reinforced plastic. There are cameras inside the counter that monitor the entire department. There are security guards, anti-slip, and all requirements are in place.*” Nurse Leader 3. The environment that is prepared correctly is the first protector of nurses, and it is also the first motivation that drives nurses to wake up in the morning and go to work with all comfort. Thus, they feel comfortable when they know that there is an environment prepared for them and can use every part of it to serve patients.

Managing the department for patients is one of the first responsibilities of the nurse leaders. They can make the environment a healthy place for nurses and their patients in order to achieve the best outcomes. For example, leaders should be flexible and cooperative with nurses in terms of choosing their days off according to their circumstances. Also, managing the department is to give every nurse a time to discuss any problems that they face. Leaders can conduct a monthly meeting to hear and solve the department's problems. If nurses feel exhausted in their current areas, they can change the department for a certain period until psychological stability returns, and then they return to it. According to head nurse 5, “*Staff may change the environment, change the place, change the department for the better.*”

#### 3.3.2. Building Teamwork

Teamwork was reported by six out of nine nurse leaders as one of the essential elements in mental health settings. Because teamwork is one of the most important factors in all healthcare facilities, healthcare providers need to work together as a team, especially when they have a shared goal which is providing the best care for their patients. Also, healthcare providers usually have different backgrounds which may affect their commutation with each other. Staff in mental health hospitals, more specifically, have to use different ways of communication in order to achieve an accurate diagnosis and best patients' outcomes which in turn may result in obtaining a healthy work environment. Having an open discussion with team is found to affect positively the relationships between team members.

Nurse leaders have the responsibility to define the way of communication that is clear for all team members. One of the participants shared that “*We do have a WhatsApp group which is found the easiest way for nurses to communicate with each other when they need especially during the work hours.*” Nurse Leader 2. Another head nurse said that “*I usually invite all staff in a weekly meeting to discuss their obstacles and the disagreements with their colleagues.*” Nurse Leader 9. Resolving the conflict between nurses in the department should be immediate to avoid any negative consequences that could affect both nurses and patients. If the issue is repeated or someone refuses to work with the other nurses, transferring nurses to another department could solve the problem.

Most of the participants shared that the relationships between teamwork is the key in mental health facilities. Nurse leaders have the role to create positive relationships between team members. According to one of the head nurses, “*The relationship must be good, so the work is shared, and the communication between us is good. Everyone is doing his job.*” Nurse Leader 1. Nurses usually face conflict every day with other healthcare providers, patients, or families. They need to resolve conflict and make their environment a comfortable place to do their jobs.

#### 3.3.3. The Required Leadership Skills in Mental Health Hospitals

Being in a position of authority is hard and requires a specific set of skills and expertise as reported by all nurse leaders. Becoming a unique leader forces them to be different and more creative in their decision-making. One of the main features of a good head nurse is to be a good listener to their subordinates, observe the issues they are facing, and try not to put too much pressure on them. Even if you do nothing to help them, they should still strive to calm them down. However, if they engage with them and listen effectively, they will feel more mentally at ease. Additionally, they must be quick in making decisions and discern whether these decisions are beneficial or not. Providing a positive impact on the nurses when they achieve something will excite them to continue their work, creating a positive atmosphere which is needed.

The observant head nurse will not try to assume the role of a leader and will instead deal with problems head-on, interacting with nurses to manage them effectively, ensuring equity, which are the most crucial aspects of this predicament. Considering employees equally and giving them constant advice on their work will keep them interested. One participant stated that “*You try as much as possible to do your job well on the basis that the employees follow your lead. Whenever the head of the department is balanced in his work, whether his administrative work or his technical work, and he has a background in all matters, this will create an attractive environment for employees, and the employee will have the opportunity to ask his boss directly because he knows that he will give him complete information about everything he needs.*” Nurse Leader 5.

#### 3.3.4. Entertainment

In the fast-paced world of healthcare, where nurses are constantly confronted with challenges and stress, the need for moments of relaxation and entertainment cannot be overstated. It is crucial for maintaining their mental and emotional well-being. As one head nurse put it, “*The nurse must certainly have a place where they can rest for 15–30 min.*” Nurse Leader 5. However, with limited space and resources, head nurses often find themselves grappling with this issue. Some have devised creative solutions, like organizing group outings or meals both inside and outside the hospital. Others prioritize creating spaces within the hospital environment itself, ensuring there is a designated room equipped with comforts like coffee and cozy seating where nurses can recharge during their breaks. Such initiatives not only foster a sense of camaraderie among the nursing staff but also promote a healthier, more supportive work culture overall.

### 3.4. Barriers to Keep the Environments Healthy

#### 3.4.1. Mental Health Nurses' Traits

Nurses, in general, have different traits and characteristics that make them different from other healthcare workers, but nurses in mental health hospitals must have more skills and features than other nurses because of the unusual environment of the mental health wards. All mental health hospitals have different needs, different circumstances, and different skills. One of the main traits that the nurse should have is looking for new courses on self-development and how to manage job stressors. Also, the mental health nurse should have physical features, unlike the other nursing departments; for example, Intensive Care Units (ICU) nurses need different and advanced skills that they learn through specific courses. Nurses in mental health hospitals depend on the physical structure unlike other hospitals. Physical structure does not matter, but here, it is important. The stronger the nurse, the better the control over the patient and the more he protects the patient from influencing factors. One of the participants said, “*We all came to help patients. Any difficulty you face you must bear for the patients. The nurse must enter the mental health department expecting the problem they're going to face.*” Nurse Leader 7.

#### 3.4.2. Burnout

Eight out of nine nurse leaders mentioned burnout in mental health settings. The causes of job burnout vary, and some of them are overlapping causes that accelerate job burnout. The causes of job burnout in mental health hospitals are related to the work environment and its lack of readiness, some of which are related to the nature of the work and direct dealing with the patient. Also, in mental health hospitals, one of the causes of burnout may be a hidden reason, which is the lack of impact on the patient. For example, a patient in surgical departments or ICU may be in a coma, but if the nurse pays direct attention to them, the patients may wake up from coma or any other patients if they were given medications and the nurses did the required care. This reflects the improvement in the patient, which reflects positively on the nurse, thus, the nurse feels that goals were accomplished. Unlike a nurse in mental health hospitals who may work twice as hard as any other nurses and does not see any improvement in patients because the patient has a chronic mental illness that may take years to recover. Thus, nurses who care for mental health patients could feel that there are no benefits from the jobs. Nurses also could feel tired and exhausted which leads to disability and finally to job burnout.

There are many solutions that nurse leaders use, on the other hand, to minimize burnout among nurses, including compensatory solutions such as vacations and reducing working hours. One of the strategies they have been using is to make nurses go through other departments to have a rest period such as outpatient clinic or home visits. Therefore, nurses can rest and stay away from stress a little, but the problem with this strategy is that the number of nurses is large, reaching 500 nurses, and the clinics are few which makes switching to these areas difficult. Additionally, there is a strategy, which is “external training,” and its idea is for the nurse to go to another hospital for training and to obtain the skills and develop their knowledge. Also, it could help to be away from the mental health hospital environment and be in the state of what is called “recharging.” The nurse who suffers from job burnout meets with the head nurse to know about the reasons that led to burnout and then transfers them to a psychologist if needed.

There are also proactive solutions such as preparing the environment by having a sufficient number of nurses in each department to manage the department “high in quantity and quality.” Thus, the nurse ensures that the nurses working with them are well qualified. One of the head nurses mentioned that “*In the event that there is a nurse who suffers from job burnout, we transfer him to the employee clinics unit, which is responsible for job burnout and has a psychologist who can deal with the employee better than the head of the department.*” Nurse Leader 6.

## 4. Discussion

The study aimed to explore the role of nurse leaders in maintaining healthy work environments for nurses in the mental health hospital. The results show that work environments in mental health hospitals should be positively built by nurse leaders in order to achieve the best outcomes related to nurse, patient, or organization. The findings, which align with Huang et al. [10], identified the mental health nursing environment as highly demanding of nurses, requiring specialized expertise. For example, patients' unpredictability often demands proper teamwork across nurses in these units. While nurse leaders have different styles of leadership, views from different participants were common, indicating it is essential for a leader to prioritize nurse safety. Chapman et al. [15] note that nurses in mental health institutions experience high physical risks such as aggression and assault. As such, nurse leaders need to prioritize the need for safe nursing stations that protect nurses. The findings reiterated the need to assess the workplace's safety by nurse leaders continuously. Creating safe places to work for nurse leaders extends to physical safety and the need to create emotional safety for nurses by coming up with strategies that reduce burnout.

The roles of teamwork in the nursing setup cannot be underestimated. The findings revealed that working in mental health environments requires high collaboration and effective communication among nurses. According to Labrague et al. [16], the complexity and sensitivity of patient care in mental health institutions and wards necessitate effective teamwork to achieve positive patient outcomes and reduce nurse fatigue. The findings revealed that nurses were more open to utilizing digital platforms such as social media for fast and effective communication across nurse teams in every rotation. Stuber et al. [17] note that nurse leaders who manage to attain proper teamwork reduce nurse job stress, fatigue, and even conflict in workplaces. Teamwork allows conflict to be settled amicably in nursing setups, allowing for better job satisfaction and reduced attrition rates. Therefore, the findings emphasize the need for nurse leaders to create teams that enable nurses to communicate effectively and express their challenges without fear. Clarity, specifically in communication across nurse teams, is key to reducing conflicts related to miscommunications.

Workplace culture is highly dependent on the leadership skills. According to Perkins et al. [18], nurse leadership styles have the potential to either build or break trust and work morale among nurses. Findings from the study revealed that participants highly appreciated nurse leaders who promoted favorable policies such as open-door policies. Specific traits such as modeling good behavior in the workplace were also highly appreciated by nurses. Participants of the study favored transformative leadership over authoritative leadership. This aligns with findings from the literature review revealing that authoritarian leadership is highly associated with low employee satisfaction and reduced job morale [16]. The findings revealed that most nurse leaders were highly proactive individuals with a transformative type of leadership. The study findings emphasized their need for empathetic leaders who were highly supportive through mentorship and vision-building.

The findings of the study printed one primary concern among nurses that nurse leaders struggled with finding a solution to, which is burnout. A leader shares their struggles in managing teams facing high levels of burnout, which is associated with high mental struggles among nurses. Findings pointed out that burnout were beyond the head nurses' control in some cases. According to Foster et al. [19], while lead nurses may attempt to control burnout, issues such as shortage of staff and repeated exposure to compassion fatigue and trauma efforts may be futile. Mental healthcare nurses also face diminished accomplishment in most cases, as outcomes in this kind of setting are not often forthright or even measurable as opposed to acute care settings. All these factors, which may be beyond the control of nurse leaders, exponentially expose nurses to burnout. However, despite the reduced control levels among nurse leaders, the study's findings showed that participants felt that regular relations and transfers across hospital units and psychological support were essential in reducing burnout. Taking time off from rotations also gave nurses time to rejuvenate and recharge. Leadership support in reducing nurse burnout is critical [19].

The study findings revealed the role of entertainment in reducing workplace stress. The study showed that nurse leaders could make mental health workplaces friendlier by incorporating entertainment interventions. Findings from the literature support the use of break rooms and team-building activities to create positive workplaces [10]. While such interventions are highly overlooked, the study found that nurses favor them, as they encourage bonding and reduce conflicts and workplace stress.

### 4.1. Strengths and Limitations

There are many strengths of this study. This study explored a phenomenon in nursing that was not discussed in depth in the previous studies. This study could help nurse leaders in mental health settings to pay attention to the importance of maintaining a healthy work environment in mental health settings. Using a qualitative study helps to explore in depth the role of nurse leaders in providing healthy work environments in mental health settings, especially given the nature and workload of these settings. On the other hand, this study has some limitations. Collecting data from one setting could affect the generalizability of this study to not be applicable to all mental health settings. Also, collecting data from only nurse leaders could avoid reaching additional results compared to if nursing staff had been included.

### 4.2. Implementations for Nursing Management

The study findings have different implications for nursing management. The findings show that nurse leaders with flexible policies, emotional intelligence, and empathy show the need to focus on building leadership capacity and efficacy. According to Perkins et al. [18], there is a need for mental health hospitals to invest in training nurse leaders with a focus on transformative leadership. If nurses are to feel safe, nurse leaders need to have a voice in creating safe workplace policies for nurses. While nurse leaders may not be able to control all workplace variables related to burnout, early detection can help create a better workplace environment for their fellow nurses. Cowman et al. [20] encourage the need for assessment tools to help nurse leaders detect early signs of any problem, allowing them to offer the necessary support to the nurses. Policies such as regular rotations, departmental transfers, and time off from rotations can help manage burnout among nurses.

Nurse leaders in mental health settings need to promote nursing reflective practices. Reflective practice allows nurses to journal their emotions and participates in peer debriefing, allowing them to preserve their mental sanity [21]. Nurse leaders need to reduce workplace stress through the reduction of conflict by creating effective teams with proper communication channels. Proper communication channels allow for peer bonding among nurses, reducing the chances of conflict and stress in mental health hospitals.

While nurse leaders bear the highest responsibility in creating favorable workplaces for the nurse employee, mental health institutions have a part to play. Issues such as staff shortages are beyond the nurse leaders' control [22]. As such, institutions must collaborate with nurse leaders by ensuring proper nurse-to-patient ratios are achieved to curb burnout. The institution also needs to empower nurse leaders by offering tailored training to nurses on the need for reflective nursing to minimize mental illnesses among nurses. Institutions need to promote wellness programs for mental health nurses and provide recharge programs that allow nurses to recharge from intense roles in the mental health units. The study highlights the need to legitimize structural support to nurses. The mental health institution must prioritize staff needs and safety. This includes utilizing regular staff supervision, increasing access to self-development workshops, and providing access to mental healthcare support. The study highlights the need for mental healthcare policies and institutions to prioritize staff nurse well-being to achieve positive outcomes. As such, institution allocations may also consider improving ward safety for nurses, increasing staff ratios, and ensuring breaks for nurses.

### 4.3. Implementations for Future Research

This study reveals the need for future research to prioritize the impact of mental health nursing policies within nursing, as they directly impact the efficacy of a nurse leader's leadership style. There is inadequate research on the need for health authorities to equip nurse leaders with leadership skills. More research is needed to increase awareness of recognizing leadership training as a competency to become a nurse leader. Further policies in mental health nursing settings cannot be similar to those of acute care units. As such, research needs to focus on identifying policies that need to formulate the standards for the mental health nursing working environment. Future research needs to focus on exploring favorable policies that include but are not limited to creating burnout assessment and prevention measures for mental health nurses. In exploring the role of nurse leaders in improving working environments for nurses, it is also crucial to explore barriers they face.

## 5. Conclusions

The findings of this study illustrated the huge role of nurse leaders in maintaining a healthy work environment for nurses. The mental health hospitals are different from other healthcare settings due to the nature of the work and environment. Nurse leaders apply different mechanisms to improve the workplace such as having open discussions with staff nurses, helping them to enhance their skills, using entertainment to change their work loads, and being flexible in their schedule. However, nurse leaders face various issues, barriers, and pressures to keep maintaining the positive environment. These challenges encompassed a range of factors, including the impact of understaffing, which increased the workload for individual nurses and contributed to elevated levels of stress and professional exhaustion. Additionally, nurses frequently encountered verbal and physical aggression from patients, and prolonged exposure to such incidents could result in physical ailments or psychological distress. This study raises a critical topic in nursing that should be emphasized in research and explores different ways that could help nurse leaders to keep the work environment healthy in mental health hospitals.

## Figures and Tables

**Table 1:** Demographics of participants.

Participants	1	2	3	4	5	6	7	8	9
Nationality	Saudi	Saudi	Saudi	Egyptian	Saudi	Egyptian	Saudi	Egyptian	Saudi
Position	Head nurse	Head nurse	Head nurse	Head nurse	Head nurse	Head nurse	Head nurse	Head nurse	Director
Age	32	50	28	42	30	40	38	40	33
Gender	Male	Male	Male	Female	Female	Female	Male	Female	Male

**Table 2:** Main findings themes.

Themes	Subthemes
Facilitators to maintain healthy work environments	Ways to maintain healthy work environments
	Building teamwork
	The required leadership skills in mental health hospitals
	Entertainment

Barriers to keep the environments healthy	Mental health nurses' traits
	Burnout

## Data Availability

Access to data is restricted due to ethical concerns and privacy of participants' information.
